# Supplementary material for: Analytic reproducibility in articles receiving open data badges at the journal Psychological Science: an observational study
Source: R Soc Open Sci. 2021 Jan 6;8(1):201494. doi: 10.1098/rsos.201494 (PMC7890505; doi:10.1098/rsos.201494)
Supplement: Supplementary Information [file rsos201494supp1.docx]

# Supplementary information

for

Analytic reproducibility in articles receiving open data badges at the journal Psychological Science: An observational study

Tom E. Hardwicke^1,2^, Manuel Bohn^3^, Kyle MacDonald^4^, Emily Hembacher^5^, Michèle B. Nuijten^6^, Benjamin N. Peloquin^5^, Benjamin E. deMayo^5^, Bria Long^5^, Erica J. Yoon^5^, & Michael C. Frank^5^

## Contents

## A. Supplementary design and procedures information

B. Supplementary sample information

## C. Supplementary results

## D. Deviations from pre-registered protocol

## E. Reproducibility vignettes

## F. Supplementary information references

## A. Supplementary design and procedures information

The most serious consequence of non-reproducibility is that it undermines the credibility of associated scientific claims. We therefore attempted to determine the extent to which any reproducibility issues we encountered had substantial implications for related conclusions drawn in the original papers. As noted in a previous investigation (Hardwicke et al., 2018), this is a complex issue that cannot be straightforwardly measured by a quantitative index. We therefore, considered this question on a case-by-case basis, using multiple factors to inform our judgement, including the presence/absence of decision errors, the quantity of and type of target values that could not be reproduced, the difference in magnitude of key values like effect sizes, and the specificity of the hypothesis under scrutiny. This was a necessarily subjective exercise; however, the judgement for each reproducibility check was agreed upon by the (two or more) team members who worked on it in addition to the first author (T.E.H.) and senior author (M.C.F.). The rationale underlying each judgement is outlined in the reproducibility vignettes in Supplementary Information E.

Determining the causal locus of non-reproducibility was not straightforward because we often did not have access to comprehensive information about the original analysis process. Additionally, there is not necessarily a direct mapping between discrete causal loci and reproducibility issues; multiple numerical discrepancies could be attributable to a single cause or multiple causes. Sometimes the causal locus of non-reproducibility became apparent after discussions with the original authors, however, all classifications were made independently by our team.

Comparisons between values were partly automated through the use of a custom R function (<https://osf.io/mqzj4/>) that accounted for rounding errors by rounding reanalysis values to the same number of decimal places as original values prior to computing the percentage error and classifying the value as a match, minor discrepancy, or major discrepancy. Note that for 212 values reported relative to a threshold (e.g., p < 0.05) or embedded in figures, we could not directly calculate percentage error and it was necessary to ‘eyeball’ (i.e., visually compare side-by-side) the values to confirm a match.

All team members had started or completed graduate level training in psychology and had experience conducting the types of data analyses typically found in an introductory psychology statistics textbook (e.g., Field et al., 2012).

In the first contact emails we sent to original authors, we explained the project, the issues we had encountered with their article, and provided a copy of an interim reproducibility report. If we were notified that the email was not delivered, we spent up to 5 minutes searching for a more recent contact email address. If the corresponding author(s) did not respond after two weeks, a reminder email was sent.

## B. Supplementary sample information

We downloaded data from the Kidwell et al. (2016) study (available here:<https://osf.io/u6g7t/>) and selected the 47 articles that had received an open data badge. Kidwell and colleagues had determined that 35 of these articles were accompanied by data sets that were accessible, correct, complete, and understandable. One team member (T.E.H.) examined each of these articles and attempted to identify a ‘substantive finding’ supported by a ‘relatively straightforward analysis’ according to the following operational definitions (adopted directly from Hardwicke et al., 2018): (A) A ‘substantive finding’ is one that supports a central conclusion of the article under scrutiny. This is to some extent subjective, but generally refers to findings supported in the article’s abstract and/or displayed in a figure/table; (B) ‘Relatively straightforward analyses’ involved behavioural data only and employed quantitative techniques that would be commonly found in an introductory level statistics textbook aimed at undergraduate psychology students (e.g., Field et al., 2012). Examples included means, medians, standard deviations, confidence intervals, standardized effect sizes, correlations, t-tests, and ANOVAs.

The first finding encountered in the article that met these eligibility criteria was selected. The target values for the reproducibility checks were a coherent set of descriptive and inferential statistics related to this finding, typically 2-3 paragraphs of information, occasionally accompanied by a table or figure. During this assessment, 9 articles were not included because a finding based on a ‘relatively straightforward analysis’ could not be identified (ID codes: 12-8-2014 PS, 21-7-2014 PS, 13-6-2014 PS, 18-7-2014 PS, 3-2-2015 PS, 6-3-2015 PS, 16-8-2014 PS, 19-4-2015 PS, 15-2-2015 PS). An additional article was not included because the data no longer appeared to be available (contra Kidwell et al., 2016; ID code: 8-11-2014 PS). In total, 25 eligible articles were identified. A bibliography file is available where article ID codes can be matched to their references (<https://osf.io/rszey/>). Note that there was a reporting error in our pre-registered protocol which said that article 16-2-2015 was not being included when it was.

A precision analysis (not pre-registered and performed after study completion) shows the range of expected margin of error (confidence interval width) for the range of possible effect sizes (proportion) given our sample size of 25 articles (Supplementary Figure 1). The maximum margin of error is 0.20.


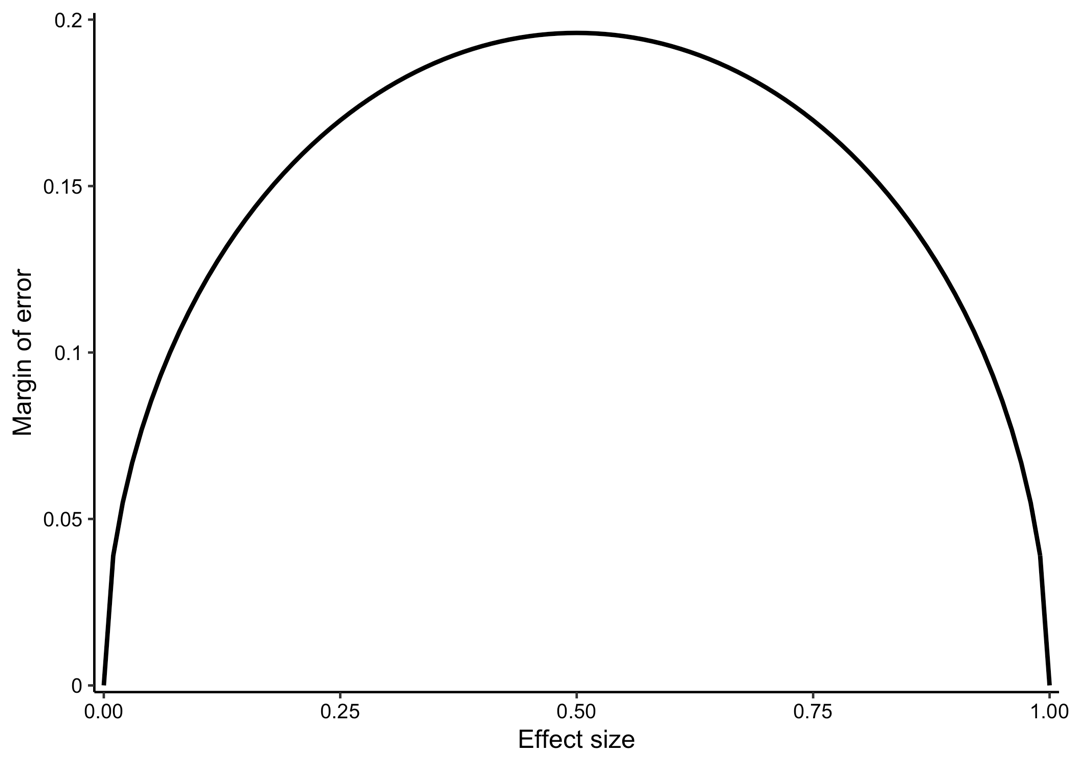


Supplementary Figure 1. Precision analysis yielding expected margin of error (confidence interval width) relative to effect size (proportion) for a sample size of 25.

## C. Supplementary results

Ninety-five per cent confidence intervals (CIs) displayed in square brackets are based on the Wilson method with continuity correction for binomial proportions (Newcombe, 1998) and the Sison–Glaz method for multinomial proportions (Sison & Glaz, 1995).

*
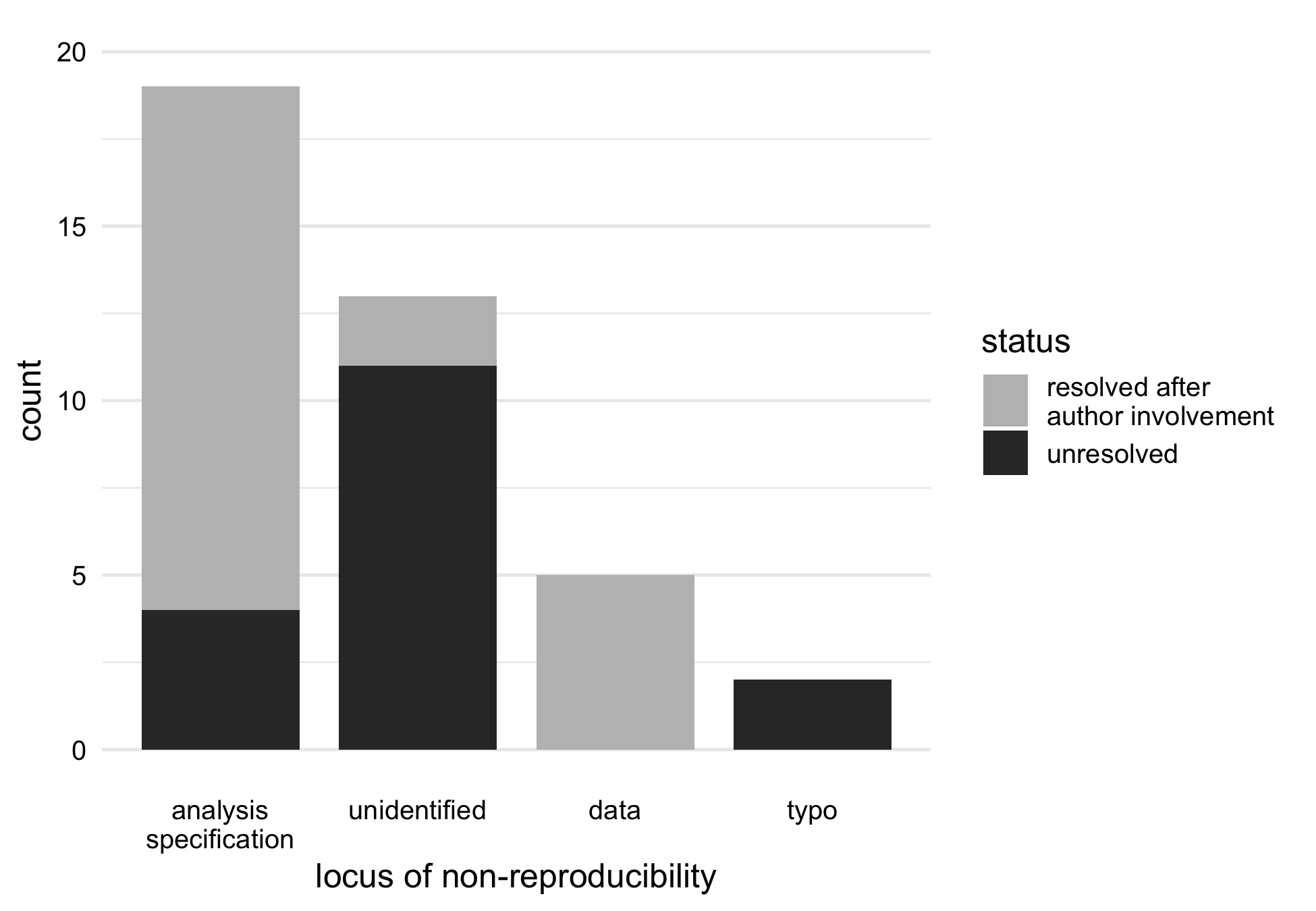
*

*Supplementary Figure* *2*. Frequency of discrete causal loci underlying non-reproducibility across all reproducibility checks. Note that some articles contained multiple causal loci.

Six articles were accompanied by analysis code, including one with Matlab scripts and SPSS syntax (8-12-2014_PS), one with Matlab scripts only (2-2-2015_PS), two with SPSS syntax only (2-10-2014_PS, 16-9-2014_PS), one with an R script (8-5-2015_PS), and one with SAS syntax (16-2-2015_PS). Three articles were reproducible without author involvement; one article was reproducible with author involvement; one article was not reproducible with author involvement; and one article was not reproducible with no author response. Team members estimated that they spent between 3 and 10 (median = 6.5, interquartile range = 3) hours actively working on each of the reproducibility checks for articles with scripts (Supplementary Figure 3).

*
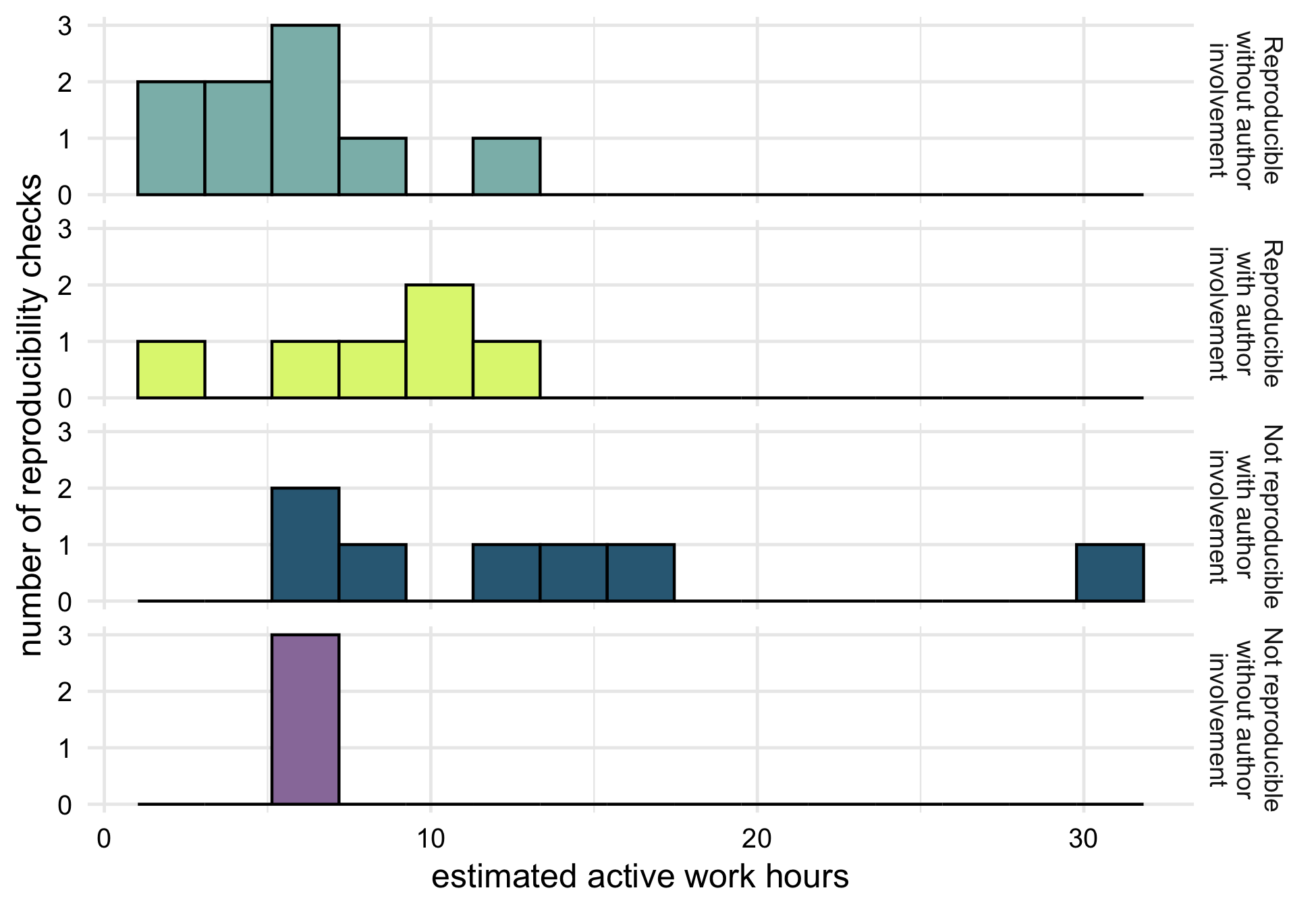
Supplementary Figure* *3*. Estimated number of active work hours spent by our team on each reproducibility check, presented separately by reproducibility outcome.

## D. Deviations from pre-registered protocol

Supplementary Table 1. Disclosure of deviations from pre-registered protocol.

| **Pre-registered protocol** (<https://osf.io/2cnkq/>) | **Final research report** |
| --- | --- |
| Does not state that we will report confidence intervals. | We decided to report confidence intervals as this would aid inferences beyond the sample. |
| Used the terminology “major/minor errors”. | To improve clarity, we use “major/minor numerical discrepancies”. |
| Used the terminology ‘reproducibility failure’ and ‘reproducibility success’ in reference to article classifications. | To improve clarity, we use “not fully reproducible” and “reproducible”. |
| Stated that “After contacting authors for assistance, a maximum time-limit of 2 months for resolution of any issues will be observed. Any reproducibility issues that cannot be resolved within this time-period will be considered reproducibility failures for the purposes of the present investigation.” | We decided not to impose this time limit as it seemed unreasonable that delays on our side could influence reproducibility outcomes. Authors were able to respond until the project was completed on June 2, 2020. |
| Does not state that we will run a meta-analysis. | When writing up the present study, we decided it would be useful to run a meta-analysis to synthesize the evidence with the study by Hardwicke et al. (2018) which is highly comparable in goal and design. |

## E. Reproducibility vignettes

Each of the reproducibility checks is summarised below in a short vignette. Each vignette contains a link to a full R Markdown report rendered in HTML that will take the reader through the reanalysis process step-by-step. A link is also provided to an OSF/Github repository that contains all relevant data and analysis code and a link to a Code Ocean container which recreates the software environment in which the original analyses were run to facilitate reproducibility.

### Vignette 1 (article 2-2-2015_PS)

Outcome: Reproducible (with author involvement)

Substantial implications for the original conclusions: N/A

R Markdown report: https://perma.cc/L86J-CWUT

OSF/Github repository: https://osf.io/ghst4/

Code Ocean reproducible analysis container: https://doi.org/10.24433/CO.4497763.v1

Description:

The authors provided extensive Matlab code outlining the original analyses and outputting what appeared to be the primary results sentences and figures in the paper. However, we found that these did not actually match up with the numbers reported in the original article. We noticed that there were some trial exclusions in the main analysis file that were described as "optional". We tried running the code both when excluding these optional trials and when including them, however, neither matched the values reported in the paper.

We asked the authors for their input. It turns out that they had already realised the original code they uploaded did not completely reproduce the reported findings, and had additionally discovered a mistake in the data they had posted to the Open Science Framework. They had already posted corrected data and code to the OSF (https://osf.io/mx92g/) but our team was not aware of this as it was not mentioned in the paper. The authors say that they had considered posting a corrigendum to the paper but decided not to pursue that because Psychological Science only publishes one if the error "significantly affects an article’s findings or conclusions or a reader’s understanding." The authors "did not feel this met that threshold".

A readme file accompanying the updated OSF materials contains further details about the original errors:

*1) The "Example analysis code" component uploaded to OSF 10/17/2014 (https://osf.io/7gvh9/) was not sufficiently documented and hard to use in replicating the published results. The code presented here is a commented version of the code used to produce the reported analyses, and therefore identically replications the results from the paper.*

*2) The data file with the mouse traces (sullivanEtAl2014ForPub_mouse.csv) contained several errors, leading to another source of discrepancy from the paper's reported statistics. The mouse tracking data uploaded in this component (newData.csv) is the original raw data.*

*3) This code reports that the percent of trials excluded from analysis is 8.1%, but the paper reports that it was 13.3%. This updated 8.1% figure is correct, and the reported number in the paper is a mistake.*

With the updated data and code, we are now able to reproduce the values and have attributed the original reproducibility problems to the errors in the data file as specified above by the authors.

### Vignette 2 (article 3-10-2014_PS)

Outcome: Not fully reproducible (with author involvement)

Substantial implications for the original conclusions: Unknown (analysis could not be completed)

R Markdown report: https://perma.cc/6FBE-GXE6

OSF/Github repository: https://osf.io/9vqs5/

Code Ocean reproducible analysis container: https://doi.org/10.24433/CO.7978743.v1

Description:

We were able to reproduce the descriptive results presented in Figure 2 but encountered problems reproducing the inferential statistics. After receiving a more specific analysis specification from the author, we were mostly able to reproduce the key 3-way interaction from the repeated measures ANOVA. Specifically, we were able to reproduce the degrees of freedom and the p-value. However, we still found a minor numerical discrepancy in the F-statistic and a major numerical discrepancy with the $\eta2$ (although the absolute magnitude of the difference was small).

Despite several attempts, we were only able to reproduce a subset of the follow-up Tukey tests and needed further information to proceed. One p-value appeared to be a decision error, because it was reported as p > 0.09 but we obtained p = 0.02.

We requested the following additional information from the authors: (1) the calculation that they used for the $\eta2$ value in the repeated measures ANOVA; (2) precisely what subsets of the data were used for each p-value reported in the follow-up Tukey tests. However, we did not receive a response to this request and have concluded that there is insufficient information to proceed with the analysis.

### Vignette 3 (article 1-1-2015_PS)

Outcome: Not fully reproducible (with no author involvement)

Substantial implications for the original conclusions: Unlikely.

R Markdown report: https://perma.cc/MY9V-97XQ

OSF/Github repository: https://osf.io/6p28a/

Code Ocean reproducible analysis container: https://doi.org/10.24433/CO.7037066.v1

Description:

We could reproduce all target values aside from one major numerical discrepancy for an F-value. In the results section of the paper it was stated that all F-values of the non-significant tests were <1, but in model 3, the F-value we obtained was 2.21. We contacted the first author on Apr 25 and both the first author and last author on May 22 to ask for their input. We’ve received no response and have therefore concluded this reproducibility check. Although the cause of non-reproducibility cannot be identified, this single observed discrepancy is unlikely to be consequential for the article's original conclusions.

### Vignette 4 (article 11-11-2014_PS)

Outcome: Not fully reproducible (with author involvement)

Substantial implications for the original conclusions: Unlikely

R Markdown report: https://perma.cc/K2Y6-58YK

OSF/Github repository: https://osf.io/huqmg/

Code Ocean reproducible analysis container: https://doi.org/10.24433/CO.3190579.v1

Description:

This reproducibility check was largely a success. There were some difficulties mapping the reported analyses to the variable names in the data file, however these were eventually resolved with a bit of guesswork.

There was one major numerical discrepancy where a p-value was reported as = .001 but we obtained 3.695211e-13. It seems very likely the intention was to report p <.001 as the reported t-value is consistent with this significance level (the authors have confirmed over email that it was probably a typo). This single observed discrepancy is unlikely to be consequential for the article's original conclusions.

### Vignette 5 (article 11-12-2014_PS)

Outcome: Reproducible (without author involvement)

Substantial implications for the original conclusions: N/A

R Markdown report: https://perma.cc/29RS-Y55L

OSF/Github repository: https://osf.io/btpnm/

Code Ocean reproducible analysis container: https://doi.org/10.24433/CO.5508100.v1

Description:

We encountered no major difficulties reproducing all target values for this article.

### Vignette 6 (article 16-11-2014_PS)

Outcome: Reproducible (without author involvement)

Substantial implications for the original conclusions: N/A

R Markdown report: https://perma.cc/USK3-244C

OSF/Github repository: https://osf.io/ubwjm/

Code Ocean reproducible analysis container: https://doi.org/10.24433/CO.2068771.v1

Description:

The inferential statistical tests employed were not explicitly named in the original article. However, we made an educated guess that they were chi-squared tests given the context, and this enabled us to reproduce all target values.

### Vignette 7 (article 1-6-2014_PS)

Outcome: Not fully reproducible (with author involvement)

Substantial implications for the original conclusions: Unlikely.

R Markdown report: https://perma.cc/89A9-QLXN

OSF/Github repository: https://osf.io/4k6v5/

Code Ocean reproducible analysis container: https://doi.org/10.24433/CO.5582627.v1

Description:

Note that for this reproducibility check, a corrigendum had previously been published for the target article, so we aimed to reproduce the values reported in the corrigendum rather than the original article. We also could not work out how to implement the inferential analyses in R and used SPSS instead - aided by the SPSS syntax provided by the original authors.

All inferential statistics could be reproduced using SPSS. However, we found one major numerical discrepancy in the descriptive statistics (which were run in R): a reported mean of 0.05, which according to our analysis was 0.005. The relevant value also seemed to be 0.005 according to Figure 1 of the corrigendum. We contacted one of the authors and they confirmed 0.005 is the correct value and the value reported in the corrigendum is a typo. This single observed discrepancy is unlikely to be consequential for the article's original conclusions.

### Vignette 8 (article 10-7-2014_PS)

Outcome: Reproducible (with author involvement)

Substantial implications for the original conclusions: N/A

R Markdown report: https://perma.cc/53BJ-BP3P

OSF/Github repository: https://osf.io/5eps7/

Code Ocean reproducible analysis container: https://doi.org/10.24433/CO.4524649.v1

Description:

We could initially reproduce all values except for one p-value reported for a simple effects analysis. After the original authors shared SPSS syntax with us it became clear that they were implementing the analysis in a different way to what we had understood from reading the paper. When we used the original method, we were able to reproduce the p-value successfully.

### Vignette 9 (article 16-2-2015_PS)

Outcome: Not fully reproducible (with no author involvement)

Substantial implications for the original conclusions: Unlikely.

R Markdown report: https://perma.cc/BD6J-U6V4

OSF/Github repository: https://osf.io/r9j83/

Code Ocean reproducible analysis container: https://doi.org/10.24433/CO.2045757.v1

Description:

We could successfully reproduce all reported descriptive statistics for the target values; however, we observed several major numerical discrepancies for correlation coefficients and their confidence intervals. However, although these cases exceeded our 10% percentage error threshold, the absolute magnitude of the difference was small (all < .03 correlation units). It is not clear to us what the source of the discrepancy is, although it could be that different software packages (the authors used SAS and we are using R) give different values for these tests.

We attempted to run the SAS program which was provided alongside the article. However, the program is trying to read in the following text files: ‘explicit.txt’ ‘sessions.txt’, ‘sessionTasks.txt’, ‘iat.txt’, which, as far as we can tell, are not shared along with the article. We contacted the corresponding author on April 23, 2019, to request these files. We initially received a response from the corresponding author on Apr 23 (2019) which offered to help in principle but noted that this could take some time. We sent follow-up emails on April 25, May 22, and July 2, but received no further response. We ultimately decided to close the reproducibility check. Note that although we made contact with an author, no substantive assistance was provided - this is reflected in the categorisation of this case as "not fully reproducible (no author involvement)"

Although we cannot identify the causal locus of non-reproducibility, the magnitude of the differences suggests that none of the issues we identified are likely to substantially change the authors' original conclusions.

### Vignette 10 (article 16-9-2014_PS)

Outcome: Reproducible (without author involvement)

Substantial implications for the original conclusions: N/A

R Markdown report: https://perma.cc/K5LB-WD45

OSF/Github repository: https://osf.io/hz8mn/

Code Ocean reproducible analysis container: https://doi.org/10.24433/CO.1533019.v1

Description:

We encountered some numerical differences for sample sizes reported in the original article compared to our reanalysis, however these differences did not meet the threshold to be classified as major numerical discrepancies. We could successfully reproduce all target values.

### Vignette 11 (article 3-4-2015_PS)

Outcome: Not fully reproducible (with author involvement)

Substantial implications for the original conclusions:

R Markdown report: https://perma.cc/PGT4-GN8U

OSF/Github repository: https://osf.io/zkmgw/

Code Ocean reproducible analysis container: https://doi.org/10.24433/CO.6468572.v1

Description:

In this reproducibility check we were able to reproduce the descriptive statistics (Figure 3) but ran into difficulties reproducing some of the inferential statistics. We contacted the authors and received assistance that resolved some issues. Specifically, information was provided about the identity of statistical tests, units of analysis, and normalization procedures, that were not stated in the paper. Unfortunately, we then lost contact with the authors and some reproducibility issues remained that we could not resolve.

We could not reproduce two effect sizes (d). The authors reported that they tried to reproduce these values themselves and could "closely reproduce the Cohen’s d values". However, they did not share the exact values they obtained in their re-analysis. We could also not reproduce one mean and one bound of a confidence interval. The causal locus of these issues is unclear.

The reproducibility issues do not appear to undermine the original conclusions. The obtained effect sizes are smaller than those reported, but not substantially so. The mean and ci discrepancies are of low magnitude.

### Vignette 12 (article 2-10-2014_PS)

Outcome: Reproducible (without author involvement)

Substantial implications for the original conclusions: N/A

R Markdown report: https://perma.cc/V4R9-V8JJ

OSF/Github repository: https://osf.io/2mq46/

Code Ocean reproducible analysis container: https://doi.org/10.24433/CO.7548371.v1

Description:

The manuscript did not specify the way that confidence intervals were computed and it was necessary to make an educated guess. All target values were reproduced successfully.

### Vignette 13 (article 3-9-2014_PS)

Outcome: Not fully reproducible (with author involvement)

Substantial implications for the original conclusions: Unlikely

R Markdown report: https://perma.cc/4PFB-LHBP

OSF/Github repository: https://osf.io/b3v7h/

Code Ocean reproducible analysis container: https://doi.org/10.24433/CO.4160541.v1

Description:

Whilst trying to reproduce an ANOVA we noted that two participants (participant 25 for both the Moroccan and Spaniard samples) had missing data for the PAST condition. However, the degrees of freedom reported in the paper appeared to reflect that the participants *were* included in this analysis. We asked the original authors for clarification and they informed us that there were typos in the data file they had posted on OSF. The wrong participant ID had been added for some lines of data, making it appear that two participants were missing data. Once the typos were corrected, the degrees of freedom in our re-analyses matched those reported in the paper.

Two major numerical discrepancies with p-values remained. In both cases, the authors reported that p = .001, but the values we obtained were lower than this. We discussed this issue with the authors and they said that it is their policy to report p-values that are <.001 as p = .001. Unfortunately, this approach guarantees inaccurate reporting. According to the APA, researchers should "report p-values less than .001 as p < .001" (APA, 2009; p. 114). The reproducibility issues we encountered do not appear to undermine the conclusions drawn in the original article.

### Vignette 14 (article 4-1-2015_PS)

Outcome: Reproducible (with author involvement)

Substantial implications for the original conclusions: N/A

R Markdown report: https://perma.cc/R2V2-Y3HS

OSF/Github repository: https://osf.io/c6u95/

Code Ocean reproducible analysis container: https://doi.org/10.24433/CO.4555168.v1

Description:

We initially could not reproduce some degrees of freedom and F-values despite trying multiple different model specifications. We contacted the authors for assistance and they correctly pointed out that we had missed an important footnote (footnote 2) which read: "The sphericity assumption for this analysis and the corresponding analysis in Experiments 2 and 3 was not met. We report multivariate test results, as recommended by Maxwell and Delaney (2004), because these tests are more optimal than correcting for sphericity. The pattern of results did not differ depending on whether we used either a multivariate test or sphericity correction." They also sent SPSS syntax and a screenshot of the output - which demonstrated successful reproduction of the target values.

By the time we received the SPSS syntax our team no longer had access to SPSS nor the expertise to independently implement these multivariate tests in R (the analysis appears to be beyond our operational definition of a 'reasonably straightforward analysis'), however the SPSS syntax and output indicates that these outcomes are reproducible.

### Vignette 15 (article 4-11-2014_PS)

Outcome: Reproducible (without author involvement)

Substantial implications for the original conclusions: N/A

R Markdown report: https://perma.cc/VA79-D42D

OSF/Github repository: https://osf.io/2kz9b/

Code Ocean reproducible analysis container: https://doi.org/10.24433/CO.2298575.v1

Description:

We encountered no major difficulties reproducing all target values for this article.

### Vignette 16 (article 5-4-2015_PS)

Outcome: Reproducible (with author involvement)

Substantial implications for the original conclusions: N/A

R Markdown report: https://perma.cc/9DEH-7YPY

OSF/Github repository: https://osf.io/bm4cg/

Code Ocean reproducible analysis container: https://doi.org/10.24433/CO.1310152.v1

Description:

Although it was specified in the paper that one participant was excluded from the analyses due to a computer malfunction, the data files provided online did not specify which participant this referred to. Thus, prior to communication with the author, we were not able to proceed with the analyses. After the author identified the participant to be excluded, we were able to reproduce all target values.

### Vignette 17 (article 6-1-2015_PS)

Outcome: Reproducible (without author involvement)

Substantial implications for the original conclusions: N/A

R Markdown report: https://perma.cc/BTS6-5MH6

OSF/Github repository: https://osf.io/f9q28/

Code Ocean reproducible analysis container: https://doi.org/10.24433/CO.0405521.v1

Description:

We encountered no major difficulties reproducing all target values for this article.

### Vignette 18 (article 6-7-2014_PS)

Outcome: Reproducible (without author involvement)

Substantial implications for the original conclusions: N/A

R Markdown report: https://perma.cc/Z8SP-CEDV

OSF/Github repository: https://osf.io/qezax/

Code Ocean reproducible analysis container: https://doi.org/10.24433/CO.0223565.v1

Description:

We encountered no major difficulties reproducing all target values for this article.

### Vignette 19 (article 7-3-2015_PS)

Outcome: Not fully reproducible (with no author involvement)

Substantial implications for the original conclusions: Unknown (analysis could not be completed)

R Markdown report: https://perma.cc/77ND-XHKN

OSF/Github repository: https://osf.io/jgp8e/

Code Ocean reproducible analysis container: https://doi.org/10.24433/CO.7977267.v1

Description:

In our initial attempts, we found a number of minor numerical discrepancies in the descriptive statistics whether we used medians or means. We then encountered major numerical discrepancies in the inferential statistics. There were a number of aspects of the original analysis and data files we were unclear about. Unfortunately, despite emailing the original authors several times we have not received a response to our questions (we have received responses saying they will get back to us, but this has not happened > 20 months after the last message). We have thus classified these issues as 'insufficient information errors'. The issues we are unclear about are outlined in full detail in the reproducibility report. In brief, they refer to unclear labels and codings for variables in the data file, unclear use of means vs. medians, unclear levels of aggregation. Despite trying out multiple combinations of these factors based on educated guesses, we remain unable to reproduce some values and cannot complete parts of the analysis and it is unclear why.

### Vignette 20 (article 8-12-2014_PS)

Outcome: Not fully reproducible (with author involvement)

Substantial implications for the original conclusions: Unlikely

R Markdown report: https://perma.cc/R38G-QQKG

OSF/Github repository: https://osf.io/5hv6f/

Code Ocean reproducible analysis container: https://doi.org/10.24433/CO.0413564.v1

Description:

We initially ran into a problem reproducing the exclusions reported in the paper. We used both the SPSS and Matlab files provided by the authors and attempted to apply the exclusions in several different ways. As all were unsuccessful, we contacted the original authors to ask for assistance. They created a video of themselves performing the analysis in SPSS and reproducing the exclusion numbers reported in the paper. Watching this video helped us to identify that we had misunderstood the sentence in the paper that reported the exclusions, specifically: "Only participants for whom both d'1 and d'2 could be computed for the analysis subset (i.e., who had non-zero counts in every cell) were included; above chance: n = 165, at chance: n = 33." We had excluded participants with missing values in any of the cells, whereas the original analysis excluded participants with missing values in cells relevant to the analysis subset. After being sent the video, we were able to reproduce this part of the analysis successfully and proceeded with the remainder of the analysis.

We were unable to reproduce two values - a t-value (0.17 reported vs. 0.12 in our analysis) and a d-value (0.03 reported vs 0.02 in our analysis). We contacted the authors again and they said they also could not reproduce these values either - in their own re-analyses they obtain the same values as we do. The reason for the differences is unknown. The authors suggest it might be due to a change in version of the SPSS software that was used to run the original analysis. Although they qualify as 'major numerical discrepancies according to our operational definition, the magnitude of these differences indicates that they are unlikely to be consequential to the authors' original conclusions.

### Vignette 21 (article 8-5-2015_PS)

Outcome: Reproducible (without author involvement)

Substantial implications for the original conclusions: N/A

R Markdown report: https://perma.cc/WN37-9T2K

OSF/Github repository: https://osf.io/uaeqw/

Code Ocean reproducible analysis container: https://doi.org/10.24433/CO.2646134.v1

Description:

We encountered no major difficulties reproducing all target values for this article.

### Vignette 22 (article 8-7-2014_PS)

Outcome: Reproducible (with author involvement)

Substantial implications for the original conclusions: N/A

R Markdown report: https://perma.cc/4779-EB7S

OSF/Github repository: https://osf.io/mxwq7/

Code Ocean reproducible analysis container: https://doi.org/10.24433/CO.7903145.v1

Description:

Initially we encountered major numerical discrepancies for the analysis of gain scores. The t-value and the effect size obtained were considerably smaller compared to the ones reported in the paper. We originally calculated the gain scores by averaging scores in each test phase (pre and post) in each condition for each subject and then subtracting the averages for the post test from the ones for the pre test. This way of calculating scores was based on the paper's description: "Gain scores were calculated by subtracting each participant’s pretest score from his or her posttest score."

We contacted the original authors and they informed us that the gain scores were calculated in a different way, namely by subtracting pre from post test scores for each specific lesson (e.g., plate tectonics) and then averaging across lesson difference scores to get a mean gain score per subject and condition. This also included excluding some lessons for some of the participants due to absence. This way of calculating the gain scores was not obvious based on the information in the paper. The supplementary material also did not include any additional information about gain scores. After correcting the way the gain scores were calculated we were able to reproduce all target values successfully.

### Vignette 23 (article 8-8-2014_PS)

Outcome: Reproducible (with author involvement)

Substantial implications for the original conclusions: N/A

R Markdown report: https://perma.cc/58C5-HWDQ

OSF/Github repository: https://osf.io/buegj/

Code Ocean reproducible analysis container: https://doi.org/10.24433/CO.2241042.v1

Description:

We initially had trouble reproducing the principle components analysis to generate the wise reasoning composite scores from the raw data due to a missing variable for question 4A from Table 1 and 5 missing values in the raw data file that were not mentioned in the paper. But after author clarifications, these issues were resolved.

Some major numerical discrepancies remained, though their absolute magnitude was low. The authors provided SPSS syntax and we were able to implement this and reproduce the values reported in the paper. Despite extensive efforts we were unable to localize the exact reason why we could not reproduce the values in R. Our suspicion is that these might be caused by opaque SPSS/R computational differences. Nevertheless, we were able to reproduce all target values using a combination of R and SPSS.

### Vignette 24 (article 9-2-2015_PS)

Outcome: Reproducible (without author involvement)

Substantial implications for the original conclusions: N/A

R Markdown report: https://perma.cc/9VCW-MXVX

OSF/Github repository: https://osf.io/cneqr/

Code Ocean reproducible analysis container: https://doi.org/10.24433/CO.7685589.v1

Description:

We encountered no major difficulties reproducing all target values for this article.

### Vignette 25 (article 9-5-2014_PS)

Outcome: Not fully reproducible (with author involvement)

Substantial implications for the original conclusions: Unknown (analysis could not be completed)

R Markdown report: https://perma.cc/SVH6-8EN6

OSF/Github repository: https://osf.io/fam6g/

Code Ocean reproducible analysis container: https://doi.org/10.24433/CO.8022934.v2

Description:

We were initially able to reproduce the descriptive statistics and the three key figures. But there were four insufficient information errors pertaining to the original analysis specifications that prevented a full reproducibility check (see reproducibility report for details). We contacted the original authors for assistance and they clarified some aspects of the analyses which resolved some issues.

However, we continued to have difficulties with the section reporting post-hoc comparisons. There were three major numerical discrepancies for p-values. The authors report using 'Sidak corrections' but it is unclear if they were applied to the alpha threshold (correctly) or the p-values themselves. If corrections were applied to the p-values, then this could potentially explain the differences, but the article does not identify the family of hypotheses for which corrections were applied. So we also cannot correct the alpha threshold. We have decided to report the major numerical discrepancies for the p-values, but also record an insufficient information error as we cannot determine whether these were decision errors or not. Additionally, for one degree of freedom, we obtained 4 whereas the article reports 3. This may be a typo but the cause is not clear.

## F. Supplementary information references

Field, A. P., Miles, J., & Field, Z. (2012). *Discovering statistics using R*. Sage.

Kidwell, M. C., Lazarević, L. B., Baranski, E., Hardwicke, T. E., Piechowski, S., Falkenberg, L.-S., Kennett, C., Slowik, A., Sonnleitner, C., Hess-Holden, C., Errington, T. M., Fiedler, S., & Nosek, B. A. (2016). Badges to acknowledge open practices: A simple, low-cost, effective method for increasing transparency. *PLOS Biology*, *14*(5), e1002456. <https://doi.org/10.1371/journal.pbio.1002456>

Newcombe, R. G. (1998). Two-sided confidence intervals for the single proportion: Comparison of seven methods. *Statistics in Medicine*, *17*(8), 857–872. <http://doi.org/cpchjg>

Sison, C. P., & Glaz, J. (1995). Simultaneous confidence intervals and sample size determination for multinomial proportions. *Journal of the American Statistical Association*, *90(*429), 366–369. <https://doi.org/10.1080/01621459.1995.10476521>
